# An Essay Read before the Medical Association of the State of Georgia, at Their Annual Meeting, at Rome, Ga., April 11th, 1860

**Published:** 1860-07

**Authors:** Robert Southgate


ATLANTA

### Medical and Surgical Journal.

Vol. V.] JULY, 1860. [No. 11

### ORIGINAL COMMUNICATIONS.

### ARTICLE I.

*An Essay read before the Medical Association of the State of Georgia, at their Annual Meeting, at Rome, Ga., April 11th*, 1860. By Robert Southgate, A. M., M. D. "*Nil remedium, nisi tempestivo usu sit.*"

Mr. President and Gentlemen—

I invite your attention to a brief examination of the following question :

The tendency to abandon the practice of general blood-letting, in the treatment of disease: Is it evidence of an advance or retrograde movement in Therapeutics?

This is a question of much interest, and worthy of the thoughtful consideration of every member of the profession.

That medical opinion strongly inclines to give up what has long been regarded as one of our most reliable means of cure, will hardly be questioned; for no very extended observation will convince us, that general bleeding has been abandoned almost, if not altogether, by many practitioners; that by a very few is it still recognized, as a safe and reliable agent in the treatment of disease.

What has led to this great change of opinion? Is it due to conviction, forced upon us by observation of injurious results from the practice, or are we merely following in the wake of a few leaders, to whose "ex cathedra" decisions the general medical mind is blindly submitting its faith? On so vital a point as the one before us, it is alike due to self respect, and the interest of humanity, that every medical man should calmly examine the grounds of his belief; for it is a fact, not very creditable to us, that we are under the influence of a strong tendency to adopt views of important points of doctrine and practice, that may be entertained and published by some leading member of the profession, without submitting them to the critical test of reason, or entertaining the question—may not such views, and the practice based upon them, be the convictions of a single mind, swayed by influences, and placed in circumstances far different from those under which we may be called to act?

The history of blood-letting, from the earliest record we have of it to the present time, would be a curious, although not very profitable, subject of study. Its origin may be traced back to a very high antiquity; for the earliest physicians, whose names have come down to us, practiced the operation. Setting aside the fabulous notion recorded by Pliny, that the idea of it was conceived from observing the habits of the hippopotamus, we will not be very far from the truth in supposing, that the benefit following the loss of blood from accidental wounds in person laboring under internal disease, as well as the salutary effects of nasal, hemorrhoidal and other hemorrhages in certain maladies, arrested the attention of observers, and led to an imitation of nature. It may be fairly presumed that, when first instituted, the practice was productive of good; otherwise, it would have fallen into disrepute; for the instinct of self-preservation is so strong that it would have recoiled from a proceeding manifestly detrimental. We find, indeed, that blood-letting, in its various forms, was practiced in all ages, and in all countries; that in the earliest times it had its advocates, and numbered its opponents; that Hippocrates, and the greater part of his disciples, espoused its cause; that Chrysippus and Erasistratus denounced it as injurious; that Celsus, Cælius Aurelianus, and Areteus of Cappadocia, were its determined friends; that Galen was its advocate, and that in his footsteps followed Paul of Egina, Alexander of Tralles, and Avicenna.—Then arose the chemical school, with Van Helmont at its head, who denounced it as injurious; and from the date of the ascendancy of the chemical doctrines to the present time, the record is anything else but gratifying to a just professional pride.

Personal animosities were engendered and mingled in a contest, which should have been decided by an appeal to the tribunals of experience and reason. We are told that Bosquillon, a Parisian physician of distinction, was so vexed at physicians, in his day, giving up blood-letting, that he practised it largely in almost all diseases, and with a profuseness shocking alike to reason and humanity; that Guy Patin, (out of spite, I suppose,) caused his son to be bled twenty-four times during the course of a single pleurisy; and that Hecquet, a physician of eminence, fell a victim to copious and repeated venesections—overwhelming evidence, certainly, of the strength of his faith, as well as of the weakness of his intellect. Such is the humiliating record of the past! We could smile at the folly of those who have preceded us, if the visions of the murdered did not rise up to rebuke any other feelings than those of sorrow and regret.

We cannot doubt that, in contrast with those who held these extreme views, there have been in all times and in all ages, physicians who discerned the golden mean, and steadily adhered to the practice of blood-letting, as an institute of nature, whose precepts they dared not despise.

Descending from those periods, the annals of which are a heterogeneous compound of truth and fable, to more modern times, we have, in the good old common-sense school of English medicine, the names of Sydenham, Baillie, Pringle, Ferriar, Heberden, and a host of others, associated with the practice of blood-letting. In our own day, it has the consistent advocacy of the sterling good sense of Watson & Billing, of London; of Graves and Stokes, of the eminently practical Dublin School of Medicine; of the sagacious Trousseau, of France, whose admirable work on Therapeutics seems to be a digest of all the learning of the Past, illumined by all the light of the Present. Hueffland, the Nestor of German medicine, after an experience of fifty years, spoke warmly in its praise; and in our own country, we have the testimony of one who, after a long life of unexampled success in the practice of medicine and surgery, bore the strongest testimony to the safety, value, and efficacy of the practice. "It may be," said the distinguished man whose words I quote, "that in some few instances, I have had occasion to think I have carried the use of the lancet too far, but I have to lament very numerous instances in which my timidity has prevented me from using it with sufficient boldness to save my patient from death." Such was the testimony of Philip Syng Physick—*Clarum et venerabile nomen.*

Until within a few years past, the medical mind seemed enjoying repose in the belief that, in general blood-letting we possessed a reliable means of cure; when, all of a sudden its equilibrium was disturbed by the startling announcement, that blood-letting was not only not useful, but positively injurious in the treatment of Pneumonia; the announcement being followed by a formidable array of statistics, which, it was confidently asserted, proved that the percentage of deaths when general blood-letting had been practiced, was largely above that under a purely expectant system of treatment. Now, this announcement must have been sufficiently startling to those who blindly accepted such statistics, as a foundation upon which could be reared the solid and well-proportioned structure of truth; but I question whether it seriously disturbed the equanimity of any one who had learned to appreciate the full and practical bearing of the simple truth, that every case of disease should, in a practical point of view, be regarded as an individual *sui generis*, to be dealt with, not in obedience to the terms of any pre-established formula, but as common sense should prescribe, after a full, calm, and deliberate estimate of all the relations and bearings that should rightfully influence and determine our mode of procedure. Let us suppose that one hundred cases of Pneumonia were submitted to treatment.—Fifty, under the purely expectant system, embracing the withdrawal of all hurtful influences, and the placing of the patients under conditions favorable to recovery; the remaining fifty being indiscriminately subjected to general bleeding, or some method of active medication; might we not very safely predict that the percentage of recoveries would be in favor of the expectant system? Let us look at this for a few moments, and see if some good reason may not be assigned for the anticipation of such a result. If there is a principle, occupying a more prominent position than any other, in the minds of experienced men of the present day, it is this—that the human system, when attacked by acute disease, institutes a movement in the direction of its own relief; and that the duty of the physician principally consists in watching the movement, aiding and co-operating with it; with this reservation—that as this movoment is sometimes boisterous and turbulent, and likely, by its violence, to defeat its end, it requires to be moderated and controlled, whilst at other times it is so crippled and oppressed, that it demands the interposition of art for its relief.

The general principle is, however, what I have stated it to be. Now, the practice of general bleeding, in each and every case of Pneumonia, seems to be in direct contravention of this principle; and when so indiscriminately employed, must lead to disastrous results, especially in such cases as are treated in large hospitals, and upon which the statistics are based. Would it not be reasonable to fear, that in many of the cases, the recuperative energies might be lowered to a point at which disease would gain the mastery, and finally achieve the victory. We know, moreover, that after a while points of stagnation take place in the inflamed tissues, and that these points must, by simple mechanical force, lead to the formation of others; and it is more than probable that general bleeding employed on the eve of the subsidence of the general excitement would promote their formation; and that cupping and blistering would then be adapted to check the progress of the more strictly localized disease. But many cases do recover after general bleeding, even in hospital practice, and some die under the expectant system; unless, therefore, we had all the data that the inductive philosophy requires, for a sound and legitimate deduction, which no statistics have ever yet given, we have no strict logical right to decide for or against either system. Under the guidance of reason and experience, we determine to leave one case almost entirely to nature, whilst in another the phenomena of oppression are so grave, or the reaction is so intense that we deem it our duty to interpose, actively, for the relief of our patient.

Whilst, then, we do not think that any safe rule of practice can be deduced from statistics, especially from hospital statistics, as applied to private practice; and whilst we should, for the reason above stated, and a strong faith in the restorative energies of the system, prefer to trust all our cases to the expectant system, rather than subject them indiscriminately to general bleeding, no such alternative is presented to the practitioner. In the independent exercise of his judgment, he can adapt his treatment to each individual case.

And is it not a humiliating reflection that in this favored epoch of man's history, with the volume of nature open before him, with the philosophy of Locke and Bacon to guide him in its interpretation, and with all the experience of the past, as a lamp to his feet, that there should be distinguished authorities arrayed on each side of this question!

Embarrassing, indeed, is the position of the young practitioner who has entered upon the duties of his mission, impressed by a due sense of responsibility. Anxious to avail himself of the clearest light to guide him through the storm and darkness, he diligently consults the latest and most approved authorities in practical medicine. For the sake of illustration, we will suppose that they differ as to the value and safety of general blood-letting in Pneumonia. The young physician is impressed by the views of the latest authority, in opposition to the practice. He is summoned to the bedside of a patient; he has diagnosed Pneumonia, and the rational symptoms and physical signs, if he has been instructed in the interpretation of the latter, proclaim the extent and gravity of the case. He is face to face with congestion and inflammation of a vital organ; and remembering a lecture in which the efficacy of general bleeding was eloquently enforced, he decides upon a resort to the lancet, but his hand is arrested by the force of the distinguished authority he last consulted, and he pursues a different and less decisive course. The patient, we will suppose, after a longer or shorter struggle with the malady, expires. The young medical man now recollects that another authority of equal distinction has declared, that in the early stage of severe Pneumonia, blood-letting was imperatively demanded; and the very natural and distressing question presents itself—Might not the result have been different, had I made a decided impression by the use of the lancet, when first called to my patient? Now the case might have terminated in death if general bleeding had been resorted to, for the result does not necessarily demonstrate the efficacy or inefficacy of the means employed; and if it could, by any conceiveble process of reasoning, be proved that the omission of bleeding had turned the scales against the patient's safety, would not the exercise of ordinary charity excuse the young practitioner, who, under a crushing sense of responsibility, had yielded his judgment to an older and should be wiser head than his own. Should not the blame rest with those who, in their writings and teachings, dogmatically proclaim that blood-letting is useful, or that it is injurious in Pneumonia, without impressing upon the mind of the pupil the simple, wholesome conservative truth—essential truth in all times, in all ages, and under all circumstances,—a truth that constituted the Pole-star of the medical worthies of former times, "*Nil remedium, nisi tempestivo usu sit.*"

But could many of us now present apply to our lacerated feelings the same balm, that might give comfort and support to the young physician under such circumstances. Fifteen, twenty, or twenty-five years have, perhaps, passed by since we commenced the great struggle against disease, suffering and death. We have learned to read the great volume of nature understandingly; to place a respectful but just estimate upon the learned disquisitions of authors and reviewers, and the eloquent lectures of erudite professors. We have had practical illustrations of the value of all our great therapeutic agents—blood-letting, cathartics, emetics, diaphoretics, tonics, stimulants, anodynes, all "*remedia tempestivo usu.*" We have learned to use them discreetly, considerately and gently, as aids to the struggling system; to control undue excitement, to solicit the return of arrested secretions, to calm and strengthen, and co-operate in the great work of Cure.

We are summoned to the bed-side of a patient; a chill has sounded the alarm of impending mischief; fever has followed; and the cough, pain in the chest, and other symptoms, call our attention to the lungs as the probable seat of disease. We place our ear to the chest, and the physical signs make the diagnosis sure; for the soft music of respiration is displaced by the crepitating, crackling and hoarse intonations of disease. We have inflammation of a vital organ pleading for relief. How shall we best administer the needed succor? It may have happened that the last case of Pneumonia under our care terminated with the life of the patient, and general bleeding had been one of the means employed. Shortly after the sad occurrence we encountered a learned disquisition, in which general bleeding in Pneumonia was declared to be injurious, as shown by the appended table of statistics, vouched for as correct. We decide to abstain from it, and trusting to some other remedy or remedies to subdue the inflammation, our patient, on the ninth day, dies, as effectually strangled as though a ligature had been cast around his trachea and gradually tightened from day to day, until the last effort at inspiration carried in scarce a thimble-full of air, and the last attempt at expiration was little more than a startling and convulsive gasp. A friend of our late patient calls to take a last look at his remains, and remarks, "how little he is changed;" and, indeed, if we should weigh the corpse we would find, perhaps, scarcely an appreciable diminution of its normal and living weight. We open the thorax! and instead of the beautiful and buoyant structures—the matchless mechanism of the Architect Divine—we find a ponderous compound of pulmonary tissues, bloody serum, mucus and lymph! Or if our patient should survive to the middle or end of the third week—the inflammation steadily advancing, hurried on, perhaps, by the brandy toddy, serpentaria and camphor, and beef tea, which the symptoms of prostration with excitement are too often recklessly thought to demand, and the odor of which as we enter the sick-room afflicts us with such a sickening presentiment of a disastrous issue—we open the thorax! and we find the same beautiful structures converted into a pulpy, softened and disorganized mass!

Now, if we have attained to that frame of mind which some medical men reach, who have been elevated to a dizzy height by the partiality of their friends, who have magnified every trifling recovery into a wonderful result of skill—a frame of mind that makes them intolerant of the suggestions of others, satisfies them that their diagnosis is infallibly correct, their therapeutics indisputably the best that could be devised, and that when their patients die, they ought to have died; if we have attained to this most unphilosophical, this most anti-progressive state of mind, we may not feel any misgivings at the sad event. But if we are humane and reflecting, and modest men, justly proud of the triumphs of our art, humbled at times under a sense of our defeats—our pride and our humility proving fresh incentives to greater dilligence in our efforts to solve the great problems of disease and cure,—might we not feel, that perhaps we had not acted altogether wisely and well?

Now, we all know that many cases of Pneumonia will recover without blood-letting, or other active treatment; that rest in bed, the removal of all sources of irritation, warm poultices to the chest, mild diaphoretic and demulcent drinks will, in time, effect a perfect restoration. But if from this we are in danger of arriving at a generalization, false—and if false, fatal—that general blood-letting is never necessary in Pneumonia, is it not our duty to pause and reflect whether the current with which the medical mind is now drifting will carry it to a safe anchorage; or whether our bark, freighted with such precious interests, is not being wafted towards shoals and rocks and deceitful quicksands, on which it may experience a disastrous shipwreck?

I have selected Pneumonia to illustrate my subject; because, if I am not greatly mistaken, the apparently formidable statistics to which I have referred led to the reaction against the remedy, and to the very natural extension of the prejudice against it, to the other inflammatory disorders. For if, in inflammation of organs so eminently vital as the lungs, blood-letting had been proved to be not only not simply useless, but positively disastrous, we must, *a fortiori*, conclude that in inflammation of organs less immediately vital, the remedy must be useless; and if useless, injurious, (for we cannot occupy neutral ground in reference to such a powerful agent as general blood-letting is admitted to be,) unless there was something peculiar in an inflamed lung that constituted it an exception. And this peculiar something pathologists have attempted to show, arguing that the exudation of lymph into the air cells of the lungs—the true seat of Pneumonia—is the very process by which the inflammation is mechanically extinguished; and as blood-letting arrests this exudation, it is therefore inexpedient in Pneumonia; and the analogy of the beneficial influence of pressure in Erysipelas of the extremities, and of strapping in acute orchitis, has been plausibly brought to bear in support of the idea. I am free to say that I do not see much force in this reasoning, but admitting that there may be some truth in this mechanical explanation, is it not likewise true that congestion and inflammation are preliminary to this process? and if, in blood-letting we have a remedy against congestion and inflammation, will it not be expedient, in the early stage of Pneumonia, to prevent this exudation, which might proceed to such extent as to interfere with that due æration of the blood upon which the vitality of the great nervous centers of animal and organic life depends.

The retrospect of my professional life brings the remedy before me as an agent of most beneficent power. Never shall I forget the signal relief I experienced whilst suffering under an attack of Intermittent Fever—a disease in which a resort to the lancet in these days would be considered little less, I suppose, than the act of a madman. In the autumn of 1844, whilst on duty as a medical officer of the army at Fort Gratiot, at the outlet of Lake Huron, a locality where Intermittents are rife, I became the subject of the disease in a Quotidian form. The difficulty in my case was an intense gastric irritability that continued, more or less, during the intermission, and, in addition, an agonizing pain in the head was constantly present. In the third paroxysm, my sufferings were so great that I sent for the only medical gentleman in the vicinity, with the view of having some blood taken from my arm, which I felt persuaded would give me the most prompt relief. The medical man arrived, and my wishes were made known to him. After making the usual examination, he declined acceding to my request. There were present none of those symptoms which were formerly considered indications for the loss of blood. There was no violent throbbing of the Carotid and Temporal arteries, no full, bounding and vigorous pulse at the wrist, no flush of the face, no injected conjunctivæ, no indications that the vis a tergo was driving into the organs a hot current of hypervitalized blood! The perverted and ataxic condition of the Ganglionic centers, and their reflected influence upon the cerebro spinal system and nerves of the heart, were antagonistic to such open and undisguised manifestations. After some persuasion, and the assurance that all responsibility should rest with myself, he acceded to my wishes. My arm was tied up, and a vein opened. Slowly and reluctantly did the blood trickle down; soon the stream became freer. then bold and salient; and after about twelve ounces had been abstracted I felt like a new man; the pain in the head, and the gastric irritability ceased during the flow, and my convalescence dated from that hour. The medical man was surprised at the result; he anticipated symptoms of sinking; he beheld relief and invigoration.

When I was assigned to the charge of the Military Hospital at the same station, in 1841, I found many of its inmates the subjects of Chronic Irregular Intermittent Fever. Shortly before my arrival I had perused the masterly paper by Macintosh, on the safety and utility of general bleeding, in the cold stage of that disease. His reasoning was plausible, and as he was a man of high character, I had no right to question his record of facts. I determined to test the practice. The patients presented the peculiar physiognomy of those who have suffered repeated attacks of the disease, and there was, certainly, nothing in their appearance that would have led one to infer that blood-letting would be of advantage. I selected several cases which had resisted the action of quinine, and showed a pertinacious tendency to return, when the remedy was suspended. In each and every case submitted to the trial, I was gratified by the result; the duration of the cold stage was lessened; the hot stage was milder, and the critical solution by diaphoresis more complete. The subsequent use of very moderate doses of quinine established the cure. In no instance was there experienced a sense of weakness; a feeling of relief and invigoration was the immediate result. The moment for closing the orifice in the vein was when the trickling was converted into a jet—the signal that reaction was at hand. Nor will it seem strange that relief should have followed the operation, when we reflect upon the crippled condition of the nervous and circulatory systems during the prolonged cold stage of an Intermittent Fever—the beautiful equilibrium of the centripetal and centrifugal forces of the system subverted, only to be restored after a long, painful and debilitating struggle. Now, doubtless, many who hear me can, in the retrospect of their professional lives, see many instances in which general bleeding was followed by the most prompt relief. If such be the fact, and it be also true that there is an increasing tendency to dispense with this important agent, is it not our duty to examine whether this tendency is in the right direction—the improvement in Therapeutics—the aim and end of all our investigations?

Seeing, then, that blood-letting is, as it were, an institution of nature; that it has received the endorsement of some of the most sagacious minds that have adorned the profession, and that our own experience bears ample testimony to its value, I might leave the subject, as having claims upon our future thoughtful consideration.

I know that a great and wholesome revolution has been effected in the science and art of medicine; for, to use the language of an elegant scholar, "How tenderly and patiently has Medicine, once so bold, aggressive and alert, learned to wait on Nature, following her hints, assisting her efforts, and relying chiefly on her own healing and recuperative powers." But revolutions in their headlong course, are apt to bury, in common tomb, the evil and false—the beautiful, the good, and the true. And as it is the duty of the political philosopher to study the rise, decline, and fall of empires, so as to derive from them maxims, for the benefit of the present and future of the race; so is it the duty of the medical philosopher to study the rise and fall of the systems, to discern the true and the false—to discard the false, to hold fast to the true, and apply it to the relief of the sufferings of our common nature. I verily believe that some of our most valuable therapeutic means are falling into unmerited disrepute; and that the future improvement of Practical Medicine will depend upon a careful review of them all,—their powers for good and evil, and their peculiar adaptation, under the cautious guidance of reason, jugement, and common sense, to the relief of the various morbid conditions we are called upon to treat.

The diseases to which, until a few years past, general bleeding was considered applicable, are Essential Fevers, in the early stage of which there was vigorous action of the heart and arteries, with symptoms of suffering in any of the great cavities; Eruptive Fevers, when there were the same signals of distress; Malarial Remittent Fevers, of a high grade, when the same signals were hoisted; Idopathic Inflammation of the Organs, contained within the cranium, thorax, and abdomen; Acute Hemorrhages; Febrile Dropsies; Severe Articular Rheumatism, with high arterial action; certain forms of Apoplectic Seizure; the various disordered manifestations due to a state of Plethora; the annoying and distressing symptoms connected with the Pregnant state; to which might be added, obstinate and unrelenting Colics with Constipation, and in Obstetrical practice, the rigid and unyielding state of the structures, through which the fætus has to be forced into a state of breathing and independent existence—an extensive field that might be re-surveyed with interest, and, perhaps, profit to us all.

Now, Continued Fevers, classed as Synocha, Synochus, and Typhus (not embracing true Typhoid Fever, which seems to be a disease of more modern origin), were the diseases in which the physicians of other days were such close and faithful observers of nature. Whilst they recognized the great truth, that, with rare exceptions, they could not be suddenly suppressed by remedial means; they did not hesitate to moderate their early violence, by venesection, cooling aperients, cold drinks, &c., and they had the satisfaction of seeing them terminate by some critical evacuation, on the seventh, ninth, eleventh, fourteenth, or twenty-first days; so rarely beyond the last period, that they were denominated Twenty-one day Fevers.

The principle upon which they acted was, that as all violent actions in the system must be followed by the reaction of prostration; and as that prostration was generally in a direct ratio to the previous excitement, it was alike the dictate of reason and common sense to diminish the excitement, as the surest means of economizing the strength, and enabling the system to effect a critical solution of the disease. I believe the principle upon which they proceeded was a sound one—sound then, sound now, sound in all time to come.

Now, what is the course pursued in many parts of our country, (and perhaps some of us have pursued it) in these Continued Fevers? We administer fifteen, twenty, twenty-five, or thirty grains of quinine in forty-eight hours, with the hope of suppressing the fever; but the fever laughs at us and our quinine. We withdraw it, for we have witnessed no good result. We leave our patient alone for ten or twelve hours, and we find at our next visit just what we might have anticipated—after the withdrawal of such a tonic as quinine—the pulse somewhat more feeble and frequent. Now commences the wine and beef-tea, and after a while, the whiskey toddy system, with, perhaps, the placebo of cold spongings, and an occasional foot bath. A diarrhea sets in, and for fear of weakness, and not reflecting whether it may be critical or otherwise, it is promptly checked; and so we go on checking the recurring diarrhea, diminishing or increasing the quantity of stimulus, occasionally putting in a little quinine; and the fever, in spite of our treatment, sometimes terminates on the fourteenth or twenty-first day; but frequently continues to the close of the fourth, fifth, sixth, and even the seventh and eighth week; and we congratulate ourselves that we have carried the patient safely through his long illness, when, I verily believe, we have most miserably thwarted the recuperative tendencies of the system, by our false method. This is strong language, but I have had some experience in these fevers, and must think that we would not have such lingering cases, if a sedative treatment were adopted in the first stage of excitement.

I am now in attendance upon a case, that illustrates the value of a sedative treatment, aided by a spontaneous epistaxis in favoring a prompt solution of fever.

I was summoned to see a little girl, five years of age, who had, previous to her attack, enjoyed excellent health. Four days before I saw her, she had experienced chilly sensations, which, after lasting several hours, were succeeded by fever, that had continued, with increasing severity, up to the morning of my visit. She was lying supine in bed, unwilling to be disturbed. Her face was flushed, and had the febrile expression; the tongue was white and inclined to dryness; temperature of the surface was elevated; the pulse numbered 130, and gave the finger applied at the wrist a sharp and angry touch; there was frontal head-ache; a careful examination detected no thoracic or abdominal trouble. I had, certainly, reasonable grounds to diagnose Continued Fever, and cautiously so expressed myself to the mother. I prescribed a solution of sulphate of magnesia with antimonial wine, to be administered every second hour, until some decided effect was produced; after which it was to be continued in diminished quantity and with longer intervals. After the administration of the third dose, the medicine was discontinued; gentle emesis had occurred twice; three copious liquid alvine evacuations followed; spontaneous epistaxis took place during the day, commencing with a jet, and trickling away for some time, until it stopped by self-limitation. At my evening visit, I found my little patient much better; the flush of the face had disappeared with the frontal headache; the cast of countenance was more natural; the pulse was soft, and numbered 120; there was a little moisture, here and there, on the surface. I prescribed a warm foot and arm bath, to be followed by a cup of warm balm tea; gentle diaphoresis and refreshing sleep followed; at my visit the succeeding morning, the patient was free from fever. The convalescence has been progressing favorably. Now, whether it was a case of continued fever or not, the prompt relief is manifest; and I cannot help thinking it fortunate for my little patient, so far as the duration of her fever is concerned, that I have not become imbued with that great partiality for quinine, in all febrile movements, entertained by my medical friends in Augusta. I have seen cases setting in, in the same manner, and in which quinine had been prescribed with the view of arresting them in their course, linger on for three or four weeks. Now, this case, it is true, does not bear directly upon the point under consideration; but if there is an arterial sedative, scarcely second to general bleeding, it is the antimonial saline solution. I took the hint from Billing, when I was a very young practitioner, and have profited by it, on many occasions, to my own great satisfaction, and the speedy and safe reduction of fever and inflammation.

From the admirable work of Wardrop, on Blood-letting, I draw the following case. In referring to the objections to the use of bleeding in Fever, and the doctrine upon which they were founded, he says: "I had an opportunity of witnessing the fallacy of this doctrine, in the case of a youth who was attacked with fever, and whom I accidentally saw, just when he was brought from school, at the commencement of the disease. He complained of a violent headache, had a flushed countenance, a typhoid tongue, a hot and dry skin, and a rapid pulse. I immediately bled him at the arm, when in the supine posture, until he fainted; ordering him a dose of James' powder every four hours, alternately with a purgative. The physician who attended the family was afterwards sent for, and in a few hours he visited the patient. When he heard the history of the case, and observed the character of the tongue, he expressed his decided opinion that the depletive system of treatment would be injurious; that the patient had all the symptoms of Typhus Fever, which would endure twenty-one days; and that it would be followed by such a train of symptoms of exhaustion and debility that, in place of blood-letting, the very opposite system of treatment ought to have been pursued. Contrary, however, to this prediction, the bleeding completely and permanently relieved the head; the skin and alimentary canal were powerfully acted upon by the antimony and calomel, and so early as the ninth day, the fever ceased." Mr. Wardrop was an eminent surgeon and general practitioner, in London. I quote the case to show that there is, after all, not such terrible danger from depleting treatment in continued fever as, in these days, we seem inclined to believe. It was in these fevers that the application of the doctrines of Brown, of Edinburgh, proved so disastrous. He divided all diseases into Sthenic and Asthenic; the latter comprising cases wherein his uniform, indivisible property of excitability, was either exhausted or morbidly increased. These fevers were classed as Asthenic, in which the excitability was exhausted and the indication of cure was, to rouse the excitability by stimulants. Rasori, an Italian physician, was completely captivated by the apparent simplicity of the doctrine, and introduced it into Italy. The occurrence of a Petechial Fever at Genoa, gave him an opportunity of practically testing its value.—The stimulant treatment produced a frightful mortality.—Seeing and acknowledging his error, he faced about, and commenced a contra-stimulant treatment, by which the mortality was vastly diminished. From that time, the Brunonian theory fell into disrepute; and it is now regarded as the most pernicious that had ever been invented, for the promotion of malicious ends and selfish purposes. Brown, you know, was at first the friend, but subsequently, the most inveterate enemy, of Cullen. In the latter were centered (if the memory of my early reading be not at fault,) all the qualifications of the popular teacher. His personal appearance was eminently prepossessing; his countenance the blended expression of benevolence, sprightliness, and intelligence; his voice was musical; his elocution fluent; his gesture graceful; his rhetoric a felicitous combination of the simple and ornate; and withal, he had achieved an enviable reputation for professional sagacity and skill. No wonder that crowds of students gathered from all parts of the world to listen to his eloquence! These shining qualities, which would have strengthened the friendship of an ingenious mind, proved too much for the poor, miserable, selfish human nature of Brown. He had gained some *eclat* by a successful assault upon a weak point of Cullen's Theory of Fever, (what theory of Fever is there that does not present a weak and assailable point?), and rendered arrogant by success, he proclaimed the dogma associated with his name. It addressed itself to a weak point in the student's mind—the desire to obtain, by a short and easy process, to that knowledge which can only be acquired by faithful and laborious investigation. His success was great, but ephemeral. Posterity has rendered a righteous verdict. Brown is now only remembered as an unscrupulous theorist; whilst Cullen still shines with a pure and steady light, in the firmament of medical literature and science.

To Broussais, among others, we owe a debt of gratitude, for having stemmed the burning current of the Brunonian doctrines, and proved that it was not from debility we had so much to fear, as from the exhausting and disorganizing power of fever and inflammation. Broussais, himself, wandered far beyond the limits of truth and safety; but his treatise on the "Chronic Phlegmasiæ," is an enduring monument of his intellectual power. And as we slake our thirst in the refreshing stream that flows through its pages, we experience a sentiment of regret, that one who gave such early promise of being a successor, worthy of the immortal author of the "General Anatomy of the Tissues," and an architect equal to the completion of the great work he had designed, should have dwindled into a visionary, one-idea enthusiast, whose common sense had, long before he was called to pay the great debt of nature, been consumed by the fires the enthusiasm of his genius had kindled. Believing, as I do, in a substratum of essential unity in all these fevers, and that their modifications depend upon epidemic and other influences, I think the same treatment is called for, but not in the same order. Sometimes the nervous sedation is so great, that early stimulation is necessary to maintain the action of the heart and arteries; whilst the presence of congestion in some of the organs may demand, at the same moment, depletion to relieve it; for, in the language of Macintosh, "it is not inconsistent with good pathology to bleed and stimulate at the same time." Then, again, the reaction is so violent in the early stage that we have to moderate it, in order to avoid secondary prostration; the subsequent treatment being either expectant, supporting, anodyne, or revulsive—according to the indications that may present themselves during their course; always bearing in mind their tendency to terminate on certain days, dated from the access of chilly sensations; which, whatever may have been the duration of the preliminary feelings of malaise, seem to indicate the day on which nature commences the movement in the direction of her own relief and restoration. If the twenty-first day should, unfortunately, have passed, without the manifestations of those signals, which indicate a favorable crisis, all other considerations must be secondary to the great principle of sustaining the forces of the system; and I cannot too strongly commend to the young practitioner, the lectures on Continued Fever, by Dr. Todd, of London, in which the "*nil desperandum*" practice is advocated, on the grounds of the most admirable and gratifying clinical results.

I conclude what I have to say upon Continued Fevers, with a quotation from Sydenham, and no one will question his thorough acquaintance with them. After stating that the invention of the term "Malignity," has been far more destructive to mankind than gunpowder, he goes on to say (speaking of Congestive Typhus): "But if it be inferred that there is some malignity in the case, not only from the purple spots, but also from finding the symptoms of Fever milder sometimes than should seem agreeable to its nature, whilst, notwithstanding, the patient is more debilitated than could be expected for the time; I answer, that all these symptoms only proceed from nature being, in a manner, oppressed and overcome by the first attack in the disease, so as not to be able to raise regular symptoms, adequate to the violence of the fever—all appearances being quite irregular. From the animal economy being disordered, and in a manner destroyed, the fever is thereby depressed, which in the true natural order, generally rises high. I remember to have met with an instance of this kind, several years ago, in a young man I then attended; for though he seemed, in a manner, expiring, the outward parts felt so cool that I could not persuade the attendants he had a fever, which could not disengage and show itself clearly, because the vessels were so full as to obstruct the motion of the blood. However, I said they would soon see the fever rise high enough upon bleeding him.—Accordingly, after taking away a large quantity of blood, as violent a fever appeared as I ever met with, and did not go off till bleeding had been used three or four times."

Upon the subject of Malarial Remittent Fever, I shall say but little. Satisfied as I am of the value and safety of general bleeding in the cold stage of Intermittents, I would not, even if my position were such as to make my recommendations worthy of notice, have any one adopt the practice.—Should it ever so happen that I should suffer from an attack in the manner recorded in the first part of this paper, I should insist on being bled—the entire reponsibility being cheerfully assumed by myself. I attended a gentleman last summer, similarly, but not so severely, affected. Remembering my own case, I was desperately tempted to open a vein; for all the usual anti-emetic routine had failed. Fortunately, Nature stepped into his and my relief. During a spell of vomiting, seemingly violent enough to have torn all the abdominal viscera from their attachments, about two tablespoons-full of red blood came up, with some mucus from the stomach; I had no further trouble with that symptom. Now, I feel morally certain, that had I opened a vein, and thereby diminished the centripetal tendency, by affording a centrifugal outlet, I would have given my patient prompt relief. But if the case had, afterwards, assumed an unfavorable aspect, a false logic would have attributed it to the bleeding; and I am sure you will agree with me, that a centrifugal movement on my part would have been not simply expedient, but absolutely necessary.

In the Formidable Remittent Fevers I encountered, during my service on the Red River in the Choctaw Nation, West, I resorted to general bleeding, to subdue the violent excitement of the circulating forces, and followed it by other sedative treatment, until the remission was so decided that I deemed the interposition of quinine expedient. I was not, at that time, acquainted with what may be denominated the suppressing power of large doses of the remedy; and although the cure was not, apparently, so prompt, it was equally satisfactory. Sometimes I found, that under the use of moderate doses of quinine, the tongue would become dry, and some tendency to delirium be manifested. I have, under such circumstances, withdrawn the quinine, recurred to a sedative treatment, and, after a while, found that its use would be followed by its admirable anti-periodic power. I know that the empirical treatment of these fevers has almost entirely superseded the rational system; but sometimes it will fail, and then we have to revert to first principles. Already is there a commencing reaction against the empirical use of quinine. Thinking men, in and out of the profession, begin to ask themselves—May it be possible that the marked increase of severe neuralgias, intractable nervous headaches and dyspepsias, may be due to its vast consumption? It is a weapon, wielded by every overseer of a plantation, and every head of a family with nearly equal skill with ourselves. Verily it is high time for the profession to look to it, lest it fall into unmerited disrepute.

We come, now, to the Eruptive Fevers, upon three of which, Measles, Scarlet Fever, and Small Pox, I will make a few remarks. These, we all know, are composed of two elements, the febrile or inflammatory, and a specific blood poisoning. I hope it will not be deemed presumptuous in me to suggest, that, at the present time, we direct our attention to the last mentioned element, to the too great exclusion of the first from our thoughts; and that we thereby throw upon the system, the duty, often oppressive, not only of eliminating the poison from the blood, but of overcoming the subacute inflammations, that are so frequently attendant upon them. No one can look upon a case of Measles, without being convinced that the poison is an irritant of a very decided character, expending its force, principally, upon the mucous membrane of the air pasages; and that the danger of the disease consists in the establishment of irritation and inflammation in those structures. Hence the physicians of the olden time did not hesitate to let blood, when the eruptive fever was high, and the pulmonary irritation great; for they knew that when the latter was very intense it would interfere with the efflorescence on the surface, which is one of the natural, and, if I may so express myself, healthy features of the disease. I had an opportunity, a few weeks since, of witnessing the salutary influence of what might be deemed excessive purgation, during the eruptive Fever of Measles.

I was called to visit a young gentleman who was somewhat alarmed by the sudden appearance on the skin of the most vivid rash I had ever seen in a case of that disease. Four days before it appeared he had experienced chilly sensations, followed by fever, hoarse cough, tender and watery eyes, and all the other symptoms that lead us to suspect the disease to be present in the system. Thinking he had taken a severe cold, he administered to himself a large dose of Epsom salts and rhubarb, (how he managed to swallow such a dose I cannot conceive,) which, for two successive days, had produced frequent liquid evacuations from the bowels. His pulse was 100, and soft; he complained of some rawness and soreness in the chest, which, however, was less than it had been the preceding day. Thinking the purging had continued quite long enough, I prescribed a Dover's powder and the use of slippery-elm tea. This was all the treatment required. The disease went through its stages, without calling for any interference. During the disappearance of the rash, a moderate diarrhœa set in, with which, as it was clearly carrying out of his system the dregs of the disease, I did not interfere. It ceased as soon as nature had completed her own work in her own way. He returned speedily to his business, without a remnant of pulmonary irritation, and with scarcely an appreciable diminution of his strength. Now the sedation of the circulating forces, by active purgation by the saline Cathartics, is scarcely second to that produced by general blood-letting. Far from interfering with the eruption, it was more vivid than any I had ever seen. I have no doubt that general blood-letting would have had an equally salutary effect, and with far less inconvenience to the patient.

With respect to the Scarlet Fever, whilst it is very clear that the poison is intensely irritant, there seems to be combined with it a septic quality which makes the practical man cautious as to the use of active sedative treatment. Experience has taught us that cases commencing in a mild manner have suddenly assumed a gravity that calls for the most devoted efforts of our skill. We see in the same family children of equally good constitution and health affected very differently; some having the very mildest form of the disease, whilst others may fall victims to putrid sore throat; and the only satisfactory way we can account for it is, (for a reasoning mind must have some explanation,) by supposing that in these last the peculiar poison of Scarlet Fever meets with some accidental element in the blood with which it forms a destructive compound. But, whether this be so or not, every practical man is cautious how he proceeds, holding himself prepared for any emergency.

I was lately called to a case that gave me some anxiety, more especially as it was the first time I had been summoned to attend the family. A little girl, aged six years, after some twelve hours of indisposition, complained of chilliness, which was followed by fever. Two spells of vomiting had taken place, followed by three dark, watery, offensive evacuations. I was summoned on the morning of the second day of its sickness. I found it with a small rapid pulse, elevated temperature of surface, tongue slightly coated, with here and there a red point showing itself; the forces were of a scarlet tint, and the breath exceedingly offensive; there was some tenderness of abdomen, on pressure. I had no difficulty in diagnosing Scarlet Fever; for in addition, there was a scarlet rash on the surface. The child had had a dark, offensive evacuation, the morning of my first visit. Thinking, from the character of the breath, and the fœtid evacuations, that nature might be expelling some deleterious matter from the system, I determined not to interfere with it.—I directed a light warm poultice for the abdomen, a mild sinapism for the throat, and toast-water acidulated with lemon-juice, for a drink. At my evening visit, I found that, after three additional evacuations, the diarrhea had ceased; the breath had lost its heavy and offensive odor. The fever being high, and the child restless, I prescribed a pleasant solution of the citrate of potassa, which was continued through the night and following day. On the morning of the third day of my attendence, finding the pulse somewhat more feeble, a small ulcerated spot on one tonsil, with enlargement of the lyphatic glands of the neck, I prescribed, as an admirable unstimulating tonic, one grain of quinine, in solution with five drops of dilute sulphuric acid, every fourth hour; a gently stimulating liniment for the throat, and a solution of chlorate of potassa with tincture of myrrh, as a gargle. But I will not tire you with the details of the treatment. Suffice it to say, that the child went safely through the disease; a result which hardly could have been expected, had I rashly checked the diarrhea, through a peurile fear of debility, and resorted either to a stimulating or actively sedative treatment.

Whilst, then, reason and experience teach us the propriety of some such course as the above, there is not wanting testimony to show that in the severe anginose forms, blood-letting and other sedative treatment is successful. I quote from Wardrop, the following case. The heroism of the practice, although it was eminently successful, seems hardly justifiable; but it shows that there is not that great danger from active depletion in Scarlet Fever which, in these latter days, we are so much disposed to attribute to it:

"A gentleman, between fifty and sixty years of age, was seized with shivering, succeded by hot skin, and febrile symptoms. On the evening of the same day, I visited him. His mind was restless and excited; his skin hot and burning; his tongue white and loaded; his pulse frequent, and the action of his heart tumultuous. His countenance was flushed, the tint of which, along with a slight redness of the throat, made me suspicious, that he had an attack of Scarlet Fever. He was immediately bled from the arm; at first the pulse rose, and a considerable quantity of blood was abstracted, before he became faint. Calomel combined with antimony, and a purgative medicine, were then given alternately every two hours. Next morning, the febrile symptoms were much subdued, but his throat had become very painful. Twenty-four leeches were now applied to the external fauces with relief. In a few hours afterward, he was again bled from the arm, a slight return of headache with fever having come on. These symptoms never returned; a fœtid slough separated from the tonsils and he was rapidly restored to health. From the decided success of the treatment, doubts were entertained of its having been really a case of Scarlet Fever; those doubts vanished, when that disease made its appearance, a few days afterwards, in two children of his family."

We sometimes lose these anginose cases; is it because the fear of debility deters us from the use of blood-letting?

As regards Variola or Small Pox, we all know, how terrible was the loss of life, under the old heating and stimulating system. A great fire occured in London, which wrapped in flames the Small Pox hospital, rendering necessary the hurried removal into the open air, during a cool night, of the miserable inmates. Humanity shuddered at the inevitable loss of life, it was thought, must necessarily result from the exposure. But how short sighted is man! that great fire taught a great lesson in therapeutics. The poor festering wretches were mostly benefited by the change.—Many recovered, who it was thought, must necessarily die. Sydenham was there, a true disciple of the Coan sage. He instantly took the hint. General bleeding, cooling cathartics, cold drinks and fresh air, were substituted for the horrible system of aromatic confections, hot stimulating drinks, red blankets and a hot stifling atmospherc, which converted the body into a great manufactory of poison, and hurried so many to the grave. Thanks to Edward Jenner, that once great pestilence is shorn of its power! and it is a lasting reproach to the profession, that the 17th of May, the day of his birth, is not celebrated in every city and town and village throughout the world. It is a day, on which wealth, and intelligence and refinement, should assemble around the festive board, to do honor to his memory. And female loveliness should be there, with its consecrating presence; for to Jenner, female lovelines owes a debt of gratitude! We have the record of a blooming young beauty, who a few weeks before, had stood before the mirror, and smiled upon the reflection of her lovely self, innocently conscious of "all those endearing young charms, "that had brought so many ardent young lovers, prostrate suppliants at her feet; a few weeks after the pitiless malady had folded her in its embrace, presenting hereself before the same mirror, and beholding the scarred and furrowed phantom of her former self, being instantly changed into a howling, shrieking maniac. We should read, now and then, the history of its ravages, its heartless immolation year after year, of thousands upon thousands; and when it did not kill, marring the human face divine, converting female loveliness into hideous deformity; a pestilence that called forth the exhibition of the best, as well as the worst feelings of the human heart; presenting, here, the spectacle of the mother, pressing to her bosom the dying child, and self oblivious, inhaling from its expiring breath, the vapour of her own destruction; and there, the spectacle of the heartless wretch, fleeing from the presence of those who had brought him into the world, in a storm of agony and distress, protected his helpless infancy, comforted him in the sorrows and disappointments of childhood, guided him gently, and affectionately, and forgivingly, through the waywardness of youth, and finally led him up to manhood! We should think of all this; and then we should turn our thoughts to the village of Berkely in Gloucestershire, England, and think of Edward Jenner, a modest, unpretending, but most able country surgeon, beloved for the amiability of his character, and admired for his unquestioned skill, taking hold of a tradition, that had been floating, immemorially, around the stable yards and dairy farms of his native county, experimenting and proving and reasoning upon it, month after month, and year after year, until he was ready to publish to the world the great generalization—falsified by any exceptional instance, I verily believe, only through the criminal neglect of his successors—that in vaccination we possessed a sure, safe and certain preventative, against the contagion of Small Pox. In a letter that has come down to us, he touchingly portrays the feelings, that at times almost overpowered him, as returning from his professional duties, he mused upon the great central object of his worldly thoughts, and the possibility of his being, under the guidance of the "Great Physician," the instrument of a mighty benefaction to his race! What a temple for the abode of true philosophy, was that man's soul! Humble, reverent, full of calm, deep thought! and as if nothing might be wanting, to invest his memory with interest, the fragrant wreath of Poetry encircled his philosophic brow; his Address to the Robin, and other pieces that have come down to us, were the emanations from a pure heart, instinct with the tenderest sensibility to all the beauties of nature. I know you will excuse this digression; it is refreshing and profitable, to dwell upon the characters of those, who, in other days, illustrated the dignity and value of our profession.

The only time I ever felt disposed to be seriously vexed with Homeopathy, was when one of its writers claimed the great discovery, as an illustration of the Homeopathic law. We see no shadow of a shade of philosophical analogy to countenance the idea. Let them take everything else from from us—sweep from under our feet the foundations of the magnificent fabric of Philosophical Medicine, and lay it prostrate in the dust—in the midst of the ruins we will cling to this; for it was a revelation vouchsafed to the visions of true genius and pure philosophy.

In acute Rheumatic Fever, with severe inflammation of the joints, it used to be the practice to take blood from the arm, not with the idea of curing the specific part of the disease by depletion, which is utterly impracticable, but with the view of moderating the general excitement and common inflammation associated with it, and preparing the system to respond to the eliminating treatment—the true treatment of the disease. Now, we all know that it would be worse than folly to attempt to cure Rheumatism by letting blood; that it would have a tendency to give a fixedness to the disease. But seeing no good and satisfactory reason why the turbulent movements of the circulation, which must, by their continuance, lead to secondary prostration, should not be moderated in this as well as in other forms of morbid action, I cannot but regard the entire disuse of bleeding as a retrogade movement, even in the therapeutics of Rheumatic Fever. Some years ago, in some medical periodical, the use of large doses of quinine was recommended as a royal road to the cure of the disease; it was tried, and some cases did get well after its use; and one medical gentleman who used it in large doses, did suppress, as it were by magic, the articular inflammation, but stood aghast at seeing his patient struck with horrible convulsions, followed by coma and death.—The proposer, doubtless, thought he was recommending something new; whereas, it was nothing more than the treatment recommended by a clear-headed English physician, by the name of Haygarth, many years before the proposer was born, who administered the Peruvian bark with a view to its invigorating action upon the nerves, which he clearly saw were implicated in the disease. But Haygarth, if I remember aright, did not recommend its use during the febrile commotion of the first stage of Rheumatic Fever. It would be interesting to know what may have been the condition of the heart, two or three months after some of these marvellous cures, by the suppressing power of large doses of quinine; whether some of the patients felt as comfortable in the cardiac region as before; whether they could ascend a flight of stairs with as little sense of palpitation and fatigue as before the attack; whether, in a word, there were not some symptoms indicating that a lesion of the central organ of the circulation might have been sustained. We must be well informed upon this point before we can endorse any such therapeutics.

The treatment by opium and the alkalies, &c., so ably advocated by Dr. Todd, of London, commends itself to us, as admirably adapted to its treatment, by its powerful anodyne and diaphoretic action—the cutaneous exhalents being the great eliminating agents by which the blood is freed from the acid elements with which it is charged. Preceded—in robust and inflammatory habits—by bleeding, cathartics, or other sedative treatment, I should think the system would be placed in the best possible condition, to be thoroughly cleared, by a continuance of the eliminating treatment, of those dregs of the disease that prove so vexatious alike to physician and patient. Dr. Todd's lecture on Rheumatic Fever, will amply repay perusal; for it is based upon the clinical experience of a clear-headed and independent thinker.

In many of the distressing symptoms of the pregnant state, general bleeding used to be employed with marked benefit. Many of us know how intractable they sometimes are, and how wretched the existence of many females becomes, almost from the very moment of conception to the end of gestation. I quote the following case from Wardrop, in illustration of the great benefit resulting from venesection:

"A lady, in a state of pregnancy, had been greatly debilitated, having vomited every kind of food and drink she had taken for upwards of twenty days. I saw her at this period. She was so emaciated and feeble that her recovery, by those around her, was considered hopeless. She had distinct tenderness, on pressure, in the epigastrium, and her pulse, which at first gave the impression of great languor, on more minute examination was very contracted, feeling like a thread, and very incompressible, whilst the heart's action was vigorous. This state of the vascular system assured me that I should afford her relief by blood-letting, which was immediately resorted to, though with hesitation, by her other medical attendants. No sooner had a few ounces of blood flowed from the vein, than the pulse began to rise and acquire volume, and upwards of twenty ounces were abstracted before its vigor was subdued. The result of this treatment was, that with very small doses of the sulphate of magnesia, repeated at short intervals, the stomach no longer rejected food, the alimentary canal was unloaded, the patient's recovery was progressive, and she was delivered of a healthy child, at the proper period."

Now, when we reflect upon the formidable dimensions that uterine pathology has assumed, within a few years past, may not a reasonable suspicion be entertained, that it may be partly due to the almost entire disuse of blood-letting, in the distressing irritations, irregular determinations and congestions of the pregnant state. In days long past, the practice was common; and our mothers and grandmothers, having fulfilled the duties of their holy mission, still live in our midst, in the enjoyment of health, the objects of our veneration, our admiration and our love. But in these days, scarcely is the young wife the second time a mother, when she presents, too frequently, a wreck of her former self. Intra-pelvic irritations and pains harrass her by day; congestions, inflammations, and ulcerations of the cervix uteri, and their sympathetic accompaniments, destroy her bloom; and we hear doctors boastlng of how often they have leeched and cauterized the delicate structures. We know, from experience, that these proceedings are sometimes absolutely necessary; that after having relieved constitutional irritation and sympathetic suffering, as far as medical treatment will answer, we are compelled to conclude that some local trouble exists, demanding local treatment. But what true-hearted gentleman is there who does not lament the sad necessity; and even when called to administer to one whose purity is swallowed up in crime, who does not wish it might be otherwise. We know that the gentlest and the sweetest of the sex, whose heart is the throne of purity, having full confidence in her medical adviser, will submit with resignation to his judgment. But if he does not feel that it is an alternative to be, if possible, avoided—if otherwise, much to be lamented—he is unworthy to minister to the infirmities of the sex. Now, may it be possible that much of this distressing ill-health is due to the fact, that we let the sufferer go through nine long months, with her headaches and flushings, her febrile movements, her intra-pelvic pains, her gastric irritations, her bloated limbs, and the other afflictions of her most interesting state, when experience has proved that the occasional loss of a few ounces of blood from the arm would sooth her troubled system, into peace and comfort. Passing by other morbid conditions, in which blood-letting was formerly practiced, I come to the subject of Inflammation, for which, in former times, it was considered, *par excellence*, the remedy.

Whatever theory we may adopt, to explain the process of Inflammation, two or three positions respecting the state of an inflamed part, have been universally admitted: First, that it contains more blood than one in a normal condition; second, that its vessels are distended or dilated; and third, that minute branches, which previously only allowed entrance to a colorless fluid, now contain colored globules.—That there is some change in the nervous sensibility of a part, anterior to all other phenomena, must likewise be admitted; all the subsequent changes being, in some way or other, related to this condition of things. The microscope has revealed to us, "that the first change in the application of a stimulus to a vascular tissue, is the momentary contraction followed by the dilatation of the artery, the flow of blood through it and the capillaries is accelerated, retardation from congestion then ensues, and lastly, stagnation at points." Let us add to this, that there is, to every inflamed part, a determination, and we have an abstract of what is really known about the process. In the language of Mr. Erichsen, "we have an increased size of the vessels, an increase in the quantity and rapidity of the blood; but conjoined with this, we have a tendency to its arrest, to its stagnation at points; and as Nature is very uniform in her processes, we have a right to infer, that in all internal inflammations we have essentially the same phenomena.

Let us now take a glance at the effects of general bleeding, in a common sense way, and see whether it may be, reasonably, brought into requisition against the process.—We open a vein, and abstract, say twenty ounces of blood. As the blood flows away, the blood-vessels slowly adapt themselves by a vital contraction, to their diminished contents; and we have the very best evidence, that this vital diminution of size takes place throghout the whole system—the inflamed parts, as well as those in a healthy condition. After the flow has continued for some time, we witness manifestations of sedation of the circulating forces, a diminution of the injecting power of the heart and arteries, unless there has been oppression, when we find the pulse to rise, indicating a more free and vigorous circulation. I have sometimes thought, in reasoning upon the subject, that the direction to take blood in a full and bold stream was faulty; except in obstetrical practice where we wish to relax the rigid and unyielding soft structures, or in obstinate constipation, when we wish to relax spasm; the delicate vessels, being, as it were, taken by surprise, and not so surely contracting on their contents, as when a more gentle flow was established. Be this as it may; can we look at the conditions of an inflamed part, as above described, and then reflect upon the effects of general bleeding, without being persuaded, that in it we possess an agent, admirably adapted to overcome inflammation? It must likewise be remembered, that general bleeding leads, secondarily, to a rapid absorption of effused fluids, which we know exist in all inflamed tissues. Now reverting, for a moment, to inflammation of the lungs, and reflecting upon the vast importance of their functions, no less than the supply of duly oxygenated blood to the nervous centers, without which their functions must soon cease, and life become extinct; is it not very reasonable to believe, that in abstaining from general bleeding in Pneumonia, we are depriving ourselves of one of the most reliable means of saving the structures from fatal disorganization?

Every now and then, the voice of lamentation comes up from some of your plantations, that fifteen or twenty valuable negroes have been carried of by Pneumonia; are we sure that oppression has not, sometimes been mistaken for true debility? Dr. Billing, in his admirable little work of 300 pages, which should be in the hands of every young practitioner, for his study by day and his meditation by night, illustrates the subject of vital oppression in Typhus, and severe inflammation of the lungs and bowels, by comparing the system, under such circumstances, "to a tired horse in a loaded cart, reaching the foot of a hill, but unable to ascend it. The stimulus of the whip may make him struggle to the attempt, but if urged, he will at length sink. If, however, some of the load be removed, he can ascend the hill, and if some of the load of blood be withdrawn, the pulse will rise as is well known and admitted in its sunken state, in severe inflammation of the lungs or bowels.

Can it be necessary in addressing such an audience as this to extend the illustrations of my subject? Shall I still farther trepass upon your attention, by picturing to you the brain, the stomach, the liver, the intestines, or the kidneys in a state of inflammation; their tissues gorged with blood, their delicate vessels distended to the bursting point, their fine network of nerves pressed upon and so irritated that they appeal successfully to the entire system for sympathy; effusions taking place, points of stagnation forming, and leading to still farther obstruction; every inflamed point, a point of determination, to which there is an increased flow; important functions interfered with or suspended. Will not this state of things, unless arrested, lead to disorganization and death, or a lingering and crippled convalescence ?and has general bleeding no power against the above described condition of things? Can it not diminish the amount of blood flowing in and to the organ, take off the injecting power of the heart and arteries, and promote the absorption of the fluids that have been effused? Can we doubt that it possesses these powers, and shall we give it up without full and due reflection!

In dealing with an inflamed extremity, we place it on an inclined plan; for what purpose? to take off the injecting force of the *vis a ter go*, and promote the returning current through the veins, and thereby relieve the limb of its excess of blood; this we cannot do in inflammation of internal organs, but is it not equally important, that they should be relieved of their excess of blood. We can cover the same extremity with cloths, moistened with cold evaporating lotions, and thereby constringe the dilated vessels, reduce heat, and diminish the inflammation. We have no such resource as this in internal inflammation; or we can bandage the engorged extremity and strap the inflamed testis, and by mechanical force extinguish the inflammation. This we cannot do with an inflamed lung or liver. In phlegmonous Erysipelas we pursue the practice introduced by Mr. Hutchinson, and endorsed by the high practical authority of Mr. Lawrence, make incisions, let out the blood and effused fluid, and save the limb from gangrene and death. We cannot deal in this way with an inflamed lung or kidney. But in general bleeding we have a remedy equal to the emergency. Shall we lay it aside without at least a deliberate reconsideration of its claims?

And how perfectly under our control is the remedy! With a finger on the pulse, and an eye directed to the expression of the patient's countenance, we at once appreciate the effect and as quick as thought, can check the flow or permit it to continue.

Other therapeutic agents will disappoint us—general blood letting rarely will. Other agents take time to effect the system; this is prompt. Guided, then, by reason, observation and experience, I will, in defiance of statistics and the infallible numerical method, open a vein, in the early stages be it remembered, of inflammation of vital or delicate organs, when I find the pulse oppressed and smothered, or tense and vigorous, persuaded that I should, by such proceeding, place the suffering organ most promptly on the road to health.

And suppose it should produce some debility, what have we to fear from it? If all the spiritual bodies of those who have departed this life since disease first attacked the race could be summoned before us, and the countless throng with one voice respond to the question—What was it that prematurely separated you from those bodies which encased you on earth, now commingled with the elements from which they sprang? Would the answer be debility? Would it not rather be fever and inflammation?

Now with reference to debility from loss of blood, will not your experience sustain me when I assert, that in a large, a very large, majority of instances, the recoveries after large losses of the circulating fluid are remarkably prompt? Will not the accoucheur bear witness to the fact? Will not the Surgeon confirm the testimony? Mr. Wardrop says, (and his experience was very great) "That having generally observed that the wounds of all those patients who had lost much blood during an operation, healed most promptly by adhesion; he was in the habit, when he operated, of letting a considerable quantity of blood flow from the smaller vessels that were divided, and he never regretted the practice."

And Professer Dugas, a few weeks since, showed me a case in which he had performed the operation of Staphyloraphy—the union of the lips of the wound being prompt and perfect, although the patient, being of the hemorrhagic diathesis, lost an amount of blood that gave the Professor great anxiety. Is prompt, perfect and beautiful adhesion of the lips of a wound, after a copious loss of blood, evidence that the lost has seriously lowered the restorative energies of the system? By one who is anxious to support a fore-gone conclusion it will be at once objected, that the prompt recovery from loss of blood, under the physiological state I have referred to, cannot be considered a fair expression of the powers of the system under acute disease. I might reply by demanding proof of the fact. Now I believe every practical man will agree with me when I assert that the states of the system that render blood letting expedient—the state of vital oppression, as admirably described in the quotation from Sydenham, and that of violent ferbrile re-action—are eminently hostile to the recuperative movements of the system.—Can any better proof of the fact be needed than that presented by the change that takes place in a wound when fever attacks the patient under treatment? We have been, perhaps, admiring observers, day after day, of the healing process in an extensive solution of continuity. The patient is attacked with fever, we remove the dressings, and find the lips of the wound that had nearly united, gaping, and instead of bland cream like-fluid, that gave such maternal shelter to the delicate and florid granulations, we find a serous unhealthy fluid issuing from the serface. What course will the medical Surgeon pursue under such circumstances? He will remove the dressings, substitute, perhaps, the tepid water dressing and use means to subdue the febrile excitement, knowing that when that is relieved the wound will recommence its process of repair. He must have been a very poor observer who has not witnessed frequent illustrations of this fact. And it will, doubtless, also be objected that when the system is suffering from the influence of a blood poison, its power of repairing the loss of blood is lowered, and, therefore, the circulating fluid should not be abstracted.

Now, is it not a truth that some of these blood poisons are intense irritants, exciting high fever and inflammations, and producing thereby prostration of power; and shall we, through fear of their influence, leave these fevers and inflammations to themselves; all our principles being swallowed up in the single idea that there is a poison to be eliminated, and that the powers of the system are to be sustained and stimulated to that end. This idea of blood poisoning is looming up into fearful proportions. It is a wonderfully convenient thing to satisfy the friends of a patient who is struggling through a long, tedious illness, its tediousness being cavalierly laid to the charge of a blood poison, whilst the sub-acute inflammations and fever are almost entirely lost sight of. Now, upon any physician who, in these days, should advocate exclusive solidism or exclusive humoralism, a commission "*de lunatico inquirendo*" should be appointed. Look at the human body—every microscopic point of the soft solids bathed in fluid, and every atom of fluid bathed, if I may so express myself, in soft solids, and how can any rational man entertain exclusive views! And then the nervous system seems at times almost to be forgotten in the Quixotic hunt after the blood poison! The nervous system, whose great office it is to receive and reflect all impressions; the system that places us in relation with the world and with ourselves; which comparative anatomy and philsiology tell us, becomes more and more complex, as we rise from the lowest form of animal life, through its various progressions up to man. The nervous system, that makes us just what we are, and which, if I may venture into the field of psychological speculation, is the material frame-work by which the spiritual body, that shall exist hereafter, is placed in existing relations with the present order of things. And as regards this blood poisoning, there are some facts that make the reflecting mind receive it with some reserve, as regards its primary operation in producing disease. A man is struck instantly dead by a stroke of lightning, or the shock of a powerful galvanic battery; We open the body and we find the brain, lungs, liver, spleen, stomach and bowels, loaded with blood, with a great deficiency of it in the cavities of the heart.

Now, in some of the worst forms of fever, that kill in a few hours, we find just the same state of things; and it is in these very fevers that the phenomena of the nervous shocks are so intense; requiring in the first instance, the most powerful stimnli to keep the patient alive. If my memory serves me, I think it was Dr. Robert Jackson, Deputy Inspector General of army hospitals in the British West Indies, who described a form of fever in which sentinels, who, a short time previous, had been posted in perfect health, were struck down, as it were, by apoplexy, which if they rallied, was followed by the most intense febrile reaction, requiring copious general bleeding to place them in a state of safety. The nervous system, I cannot help regarding as the recipient of the first morbid impression—the starting point, from which proceed all the subsequent phenomena of fevers. Now do not suppose that I ignore the pathology of blood poisons. I know there are some so virulent that, under their influence, the whole system seems ready to crumble into ruins, and that we dare not use lowering treatment. But when it takes such possession of the mind as to prevent us from using sedative treatment in the earlier stages of continued fevers, I cannot help regarding it as exercising an influence out of all proportion to its intrinsic importance. If the medical history of the past teaches any truth in reference to the future there is a destined reaction to take place in medical opinion. Another petechial fever may break out, in which indirect will be mistaken for direct debility. Another Rasori, captivated by the simplicity of the doctrine, will resort to stimulation, and be awe stricken by the frightful mortality. Is it not the duty of each one of us to give this subject a thoughtful reconsideration?

The object of this paper is to encourage a review of the subject of General Bleeding as a means of cure, and not to thrust my views upon you as worthy of adoption. We have met together for mutual improvement; and if each member of the Association would, at each annual meeting, place his offering on the altar, can we doubt that it would promote the great end and object of our union. Let not the youngest member withold his contribution through fear it might not be received with fraternal consideration and respect. Like the widow's mite, it might seemingly be the least of offerings; but flowing from heartfelt devotion to truth and science, it might prove of more real value than all the rest; like the grain of mustard seed, the least of all seed, it might contain within itself the germ of a beautiful tree, under the future shadow of whose leaves and branches many a disease-striken sufferer may repose in peace and comfort.

I am well aware that, as general bleeding is an agent of great power for good so must it be for evil; and that it should be used under the guidance of a sound discretion; that age, and sex, and temperament, and previous state of health, and prevailing epidemic influence should all be duly weighed and estimated. But as there is a strong tendency of the medical mind to run into extremes; may it not be that we are verging to a point that may terminate in total negect of the practice.

Our justly placed confidence in the powers of quinine, doubtless has had something to do with the disuse of blood-letting. No one sets a higher value on it than I do. I have tested its powers from the great lakes of the North to the San Saba river in Texas, and from regions far beyond the Mississippi, to the shores of the Atlantic, and have been rarely disappointed. As the great tonic and anti-periodic, how matchless are its powers! Even in inflammations, with what advantage may it be applied, when we detect a paroxysmal increase of general excitement, which must re-act unfavorably on the original disease.

Powerless, with a rare exception, to arrest the march of a Continued Fevei, how admirably does it act in the latter stages of the disease, when administered in moderate doses in solution, in a form acceptable to the stomach, and easy of absorption; being circulated to the organic nerves of the delicate blood-vessels, (for I will not dishonor the remedy by supposing that it has an elective tendency to any other than the nervous tissue) giving them tone, and thereby bringing back the languid capillaries to a state of health; co-operating with the silent yet ever-acting energies of the system, in the direction of its health and safety. And when administered in doses proportionate to the threatened emergency of one of those terrible chills which sometimes extinguish life, with what commanding power does it step in, taking hold of the organic nervous system, as it were, bodily, lifting it up to an elevation, at which the dreaded sedation becomes well-nigh a vital impossibility, and holding it there, with a steady hand, until the great crisis has past! Yet, with all its power, can we rightly and justly regard it as a substitute for blood-letting, in those terrible inflammations, that threaten disorganization and death?

And may we not have been somewhat influenced to its almost total neglect, by the popular current against it, set in motion by a learned but visionary German enthusiast? I do not mention the system of Hahnemann, to revile or denounce it; the cause of truth never was advanced by such a course. We should not judge of it, as professionally represented by many of those whose ignorance, arrogance, and pretension affect us with sentiments of loathing and disgust. There are intelligent and worthy gentlemen who have professed conversion to its faith; and ordinary courtesy demands that we should give them credit for honesty and truth. We do not comprehend how it is possible that one, thoroughly educated in the science of his profession, and who has experienced the triumphs of its art, can abandon its well-tried resources—No! We have seen the flag of regular medicine floating triumphantly over too many battle-fields, to lower it at any such bidding; but there may be some truth in the system, and if so, is it not our duty to examine, test, and find it, and when found, apply it for the benefit of our race? Acquainted as we are, with the natural history of disease; cognizant of the restorative powers of the system, witnesses at the bedside, at which we have sat, anxious, yet patient, observers of the struggling system, of sudden improvements, which, had they been preceded by inert medication, a puerile logic would have attributed to it; we surely are qualified to discern the true from the false. It is our duty, then, to examine and test the system in the spirit of candor, and if there be truth in it, to use it for the relief of suffering. If we should so act, I think the occupation of many would be gone; for those with whom we have smiled in the sunshine, and sympathized in the cloud, from whom we have often received the tribute of greatful hearts, who have, perhaps, addressed to us the touching appeal, "Doctor, we know that you have done all that man can do, we know our child must die, but it will be a comfort to us to have you near until the dreadful agony is past," would hardly give us up for strangers, who have no such claims upon their hearts.

There is an impression on the medical mind, that, for some years past, we have been in a cycle of influence that has rendered the system intolerent of depletion. If so, will it last always; have we had any evidences that we are approaching the circumference of a different cycle, during the revolutions of which, a more anti-phlogistic medication may be demanded? As faithful sentinels, is it not our duty to be on the alert, so that we may not be surprised by a sudden onset of the enemy?

However this may be, the question I have so imperfectly discussed in your hearing, is worthy of calm consideration. I lean to the opinion, that we are making a retrogade movement in the growing tendency to give up general bleeding in the treatment of disease. You may differ with me, for honest differences of opinion will exist as long as we are what we ought always to be, free and independent thinkers. But there is one point on which we can unite in full communion of spirit and feeling—the advancement of our profession. Assembled together from different sections of this great State; leaving behind our professional joys, sorrows, hopes and disappointments, and I trust all unworthy feelings; let us register a common vow of future devotion to its honor; and when we consider the precious interests intrusted to our care, is it not worthy of our most faithful study? Whenever we cease to realize the great responsibility that rests upon us, we are worthy no longer, to minister at its altars. Let us then, for the future, so study and practice our profession that it may be exalted in the estimation of the world; and be still more worthy of the graceful tribute paid to it by the Roman orator, as it was illustrated in the person of his physician and friend.

"*Homines ad deos nulla re proprius accedunt, quam salutem hominibus dando.*"

